# Firearm experiences and safe storage challenges among a sample of Black adults: a rapid qualitative analysis

**DOI:** 10.1186/s40621-025-00634-5

**Published:** 2025-11-24

**Authors:** Evan V. Goldstein, Aryanna Sanger, Jennie L. Hill

**Affiliations:** 1 Department of Population Health Sciences, Spencer Fox Eccles School of Medicine, University of Utah, 295 Chipeta Way, Salt Lake City, 84108 UT USA; 2 College of Social Work, University of Utah, Salt Lake City, 84112 UT USA

**Keywords:** Public health, Black or african american, Firearm

## Abstract

**Background:**

Firearms are a leading cause of death among Black adults in the U.S., accounting for more than 300,000 years of potential life lost in each of the past five years. Firearms are particularly challenging for suicide prevention in Black American communities. Few recent studies have been dedicated to investigating firearm ownership and safety challenges among Black adults. Without this understanding, promising interventions such as safe storage programs and lethal means counseling may inadequately address the specific challenges and motivations relevant to Black firearm owners. Our objective was to gather feedback from Black adults with access to firearms in their homes on their (1) experience with firearms (2), rationale for firearms being in their homes, and (3) perceived challenges or barriers to safely storing firearms in their homes.

**Methods:**

This study involved semi-structured interviews and rapid qualitative analysis. 15 Black adults ages 18 years and older in the U.S. who had access to firearms in their homes participated. We developed a Transcript Summary Template to deductively condense all relevant information for three topics. Matrix analysis techniques were used to identify emerging key concepts in the responses to each topic.

**Results:**

The participants’ relationships with firearms appeared to be shaped by family traditions, vulnerability, and safety concerns that sometimes extended across generations. Participants described various pathways to firearm access, from childhood introductions through parents who viewed firearms as necessary protection against racial threats, to adult acquisition motivated by experiences of violence, discrimination, or a hobby. Key challenges to household firearm storage safety emerged: limited access to firearm safety knowledge and training in Black communities, the financial and time costs associated with safe firearm storage, and concerns about children gaining access to firearms and the importance of communicating with children about firearms in the home.

**Conclusions:**

This study provides new insights into factors shaping firearm ownership and perceived challenges to safe firearm storage among a sample of Black adults in the U.S. Our findings may help inform community-driven and clinician-focused initiatives that acknowledge the context of Black firearm ownership while promoting evidence-based safety practices.

## Background

Firearms are one of the leading causes of death among Black adults in the U.S., accounting for more than 300,000 years of potential life lost in each of the past five years [1]. In 2023, the combined rate of homicide, suicide, and unintentional deaths by firearm among Black adults ages 18 years and older (35.24 deaths per 100,000 persons) was greater than the mortality rates for many other causes of death except, for example, heart diseases, malignant neoplasms, accidents, cerebrovascular diseases, and diabetes mellitus [1, 2]. Firearms are particularly problematic for suicide prevention in Black American communities. In 2023, firearms accounted for about half of suicide deaths overall in the U.S. but nearly 6-in-10 suicide deaths among Black adults [1]. Since 2010, rates of suicide death involving firearms among Black adults have increased by 88.4% (i.e., from 3.53 to 6.65 deaths per 100,000 persons in 2023) [1]. Over the same period, rates of suicide death involving firearms among Black adolescents ages 10–19 years increased by over 250% (i.e., from 0.93 to 3.28 deaths per 100,000 persons in 2023) [1]. 

Although firearms alone do not cause individuals to become suicidal or homicidal, numerous studies have demonstrated significantly higher odds of both suicide death and homicide victimization among persons with access to firearms in their homes compared to those without access to firearms [3]. As outlined in the 2024 *National Strategy for Suicide Prevention*, the secure storage of lethal means – such as storing firearms locked and unloaded and ammunition separately in the home – is a promising intervention for suicide prevention [4]. Specifically, one older study found that storing household firearms as locked, unloaded, or separate from the ammunition was associated with lower risk of unintentional firearm injuries [5]. More recently, researchers have estimated that up to one third of youth firearm deaths could be prevented by families safely locking the firearms stored in their homes [6]. 

There are various known reasons for owning firearms, such as the need for self-protection, recreation/hunting, and rights/activism [7, 8]. Analyses of data from national surveys have suggested that some Black firearm owners may be characterized as primarily owning firearms for self and family protection [8], while one recent study of a large sample of Black adults from a selection of states demonstrated that exposure to discrimination may be associated with firearm ownership [9]. Such findings appear to be consistent with studies describing the motivation of Black adults who have experienced firearm injuries to possess firearms as a response to the fear of experiencing violence in their communities [10]. In particular, White et al.’s (2023) study of predominantly Black firearm carriers in an area of Brooklyn, New York, experiencing high rates of interpersonal violence demonstrated that over 70% of the study’s participants carried firearms out of fear for their lives or the lives of family members [11]. Regardless of the reasons for keeping firearms in the home, previous studies have also estimated that many – if not most – households store their firearms unlocked and/or loaded [12, 13], and that many Black firearm owners are unlikely to use safety mechanisms like gun safes and locking devices [9]. 

Despite recent evidence suggesting increases in firearm ownership among Black adults over the past five years [14–16], few recent studies have exclusively investigated the depth and nuances of safe firearm storage challenges experienced by Black adults [9]. This knowledge gap is problematic because firearm ownership motivations and safety practices are likely shaped by distinct community contexts and experiences, with Black adults potentially navigating different personal protection concerns and cultural perspectives on firearms than other demographic groups [17]. Without this understanding, promising interventions such as safe storage programs and lethal means counseling (which may improve safe firearm storage practices [18]) might inadequately address the specific barriers and motivations relevant to Black firearm owners, potentially limiting their effectiveness in promoting household firearm safety or even saving lives within Black communities. This gap also challenges suicide prevention initiatives in healthcare settings, such as the Zero Suicide framework [19], which strives to identify all people at risk of suicide but may miss or inadequately serve Black patients without understanding the specific firearm-related contexts and concerns within their communities.

To address this knowledge gap, our objective was to gather feedback from Black adults with access to firearms in their homes on their (1) experience with firearms (2), rationale for firearms being in their homes, and (3) perceived challenges or barriers to safely storing firearms in their homes.

## Methods

### Study design

This was a basic qualitative study involving semi-structured interviews and rapid qualitative analysis [20–23]. The University of Utah Institutional Review Board (IRB) approved the study protocol (IRB_00169397).

### Participant recruitment

Black adults ages 18 years and older in the U.S. who had access to firearms in their homes were eligible to participate in the interviews. Participants were recruited using a combination of purposive and snowball sampling techniques, including word-of-mouth referrals from participants who completed interviews, study advertising shared electronically with chapter affiliates of the National African American Gun Association (NAAGA) and Black-owned gun clubs/businesses, and Study Locator, a recruitment tool to help inform the public about research opportunities [20, 24, 25]. All participants were provided an informed consent cover letter approved by the University of Utah IRB, and verbal consent was confirmed prior to beginning each interview.

Recruitment continued until (1) the expected range of participants was met and (2) information saturation was deemed satisfactory. Given the sensitivity of the topic of firearm mortality in Black American communities, we anticipated that participation may be limited. As such, through our grant funded by the National Institutes of Health, we had a preestablished participation goal of 10 to 15 interviewees. Information saturation was then considered using a new information threshold of 5% or less per interview topic. The 5% threshold was based on Guest et al.’s (2020) method for assessing saturation [26], and is a threshold that has been noted in recent studies on firearm safety (e.g [27]). We used a base set of 9 interviews, as Hennink and Kaiser (2022) suggest that saturation is typically achieved after at least 9 interviews [28], and a run of each successive interview from 10 to 15 [26]. For each topic, we retrospectively calculated and present the quotient of new, relevant ideas documented in the transcript summary (described below) of the final interview divided by the sum of unique, relevant ideas documented in the base set of transcript summaries. For their time, each participant was provided compensation valued at $50.

### Data generation

Semi-structured interviews were conducted by the Principal Investigator (EG) from May to October 2024. An interview guide was developed, piloted, and finalized prior to conducting the interviews. The interviews contained six core questions. The present analysis focuses on three open-ended questions to elicit participants’ (1) experience with firearms (2), rationale for firearms being in their homes, and (3) perceived challenges to safely keeping firearms their homes. The remaining questions were conceptually distinct – concerning perceptions on firearm safety counseling and injury prevention – and the subject of a companion paper. Open-ended questions were used to create space for participants to share their authentic perspectives and experiences in their own words and uncover unanticipated responses [29]. Probes were used as needed to clarify ambiguous responses and encourage participants to expand on brief answers (e.g., “Can you say a bit more about that?” “This may include challenges to storing your ammunition separately from your firearm, storing your firearm in a gun safe, or other barriers?”). The interviews were conducted telephonically to reduce barriers to participation and audio recorded with participant consent. To enhance trustworthiness [20], EG examined his influence in this process through constant self-reflection and reflexive memo writing [30]. The purpose of these activities was to reflect on and limit any potential biases from influencing how he interacted with the participants (e.g., the use, wording, or timing of probes; mindfulness regarding his reactions) or affecting the participants’ responses. For reference, EG’s positionality as it relates to this topic has been described elsewhere [31]. 

### Data analysis

The interview audio recordings were professionally transcribed and reviewed by the interviewer for accuracy. We followed established steps for rapid qualitative analysis [21, 22]. First, we drafted three topics, each directly mapped from one of the open-ended interview questions. The purpose of the topics was to keep the analysis closely tied to the questions (and thus the study objective) in a clear and organized manner, allow the research team to efficiently summarize the interview transcripts for relevant information, and facilitate comparison across participants. Through discussion, we finalized each topic name to ensure it captured the main idea of its corresponding question. This was done to improve consistency across the multiple authors who summarized the transcripts. Table 1 shows the interview questions and corresponding topic names.

Second, we created a Transcript Summary Template. The template contained space to deductively condense and document all relevant information shared by each participant for each topic. Third, to pilot and calibrate the Transcript Summary Template, two authors (AS and EG) summarized three interview transcripts. Discrepancies were discussed and resolved by consensus to ensure (1) all relevant information for each topic was identified and (2) the topics were well understood and named before summarizing the remainder of the transcripts. Fourth, one author (AS) summarized the remaining transcripts, while a second author (EG) reviewed each additional summary for accuracy using the original transcripts. Transcript summarization was done at a semantic level (i.e., describing relevant information provided by the participants rather than interpreting it) [32]. 

Finally, two authors (EG and AS) used matrix analysis techniques to identify emerging key concepts in the responses to each topic. As opposed to fully developed themes, the key concepts reflect early patterns observed across participants and serve as a useful foundation for collaborative team interpretation and dissemination. This entailed inputting all information included in the transcript summary documents into one matrix with individual columns by topic and rows for each participant. Then, by topic, EG and AS reviewed the matrix for recurring patterns across the summarized, relevant insights; consolidated the recurring concepts into a separate page; and discussed and named each key concept [33]. EG and AS shared positionality statements, wrote reflexive memos, and met weekly as they summarized the transcripts and compiled the matrices. The purpose of the reflexive activities was to acknowledge their personal thoughts and reactions during data analysis, reflect on potential biases, and limit their positionality from influencing how they interpreted meaning from the subjective truths experienced by the participants.

**Table 1:** Interview questions and topics

Interview guide question	Topic
1. Could you tell me a bit about your experience with firearms?	1. Experience with firearms
2. Can you tell me about why you own a firearm or live in a home with a firearm?	2. Why firearms are in the home
3. Can you tell me about any specific challenges or barriers you experience that prevent you from safely storing firearms in your home?	3. Challenges to keeping firearms safe in the home

## Results

We interviewed 15 Black adults in the U.S. who had access to firearms in their homes. The participants were 40 years old on average (Table 2). Ten (67%) of the participants were male, and 5 (33%) were female. Geographically, 10 states (CA, FL, GA, IL, LA, MA, MI, NC, UT, TX) and all U.S. Census Regions were represented by the participants. On average, the interviews lasted 44 min. 3.2%, 5.8%, and 4.8% of the final participant’s relevant insights were deemed new information for Topics 1, 2, and 3, respectively. This pattern suggests little new information provided by the final interview in response to the three questions.

**Table 2:** Participant characteristics (*n* = 15)

	Mean/ frequency	Standard deviation/ percent
**Age in years, mean, SD**	40.3	10.6
**Sex, n, %**		
Female	5	33.3%
Male	10	67.7%
**Race, n, %**		
Black/African American	15	100.0%
**Census region, n, %**		
West	3	20.0%
Midwest	2	13.3%
Northeast	2	13.3%
South	8	53.3%

### Topic 1: experience with firearms

#### Key concept 1.1: early introduction to firearms through parents

Most participants were introduced to firearms at an early age, often by their fathers or mothers. The participants’ parents commonly kept firearms in their homes for safety and protection, motivated by fears of racial vulnerability as Black individuals in a majority white society, perceived political threats (e.g., during the Cold War), or exposure to firearm violence in their communities. Firearms were also kept for other reasons, such as parents’ being introduced to firearms through military careers, recreational use and fun, hunting, rural living (e.g., the necessities of living on a farm), and as a shared family hobby – especially between fathers and their children, e.g.:

My father used to take us to the shooting range. There used to be a police range. We’d go shoot and then, after the police range, after shooting, we’ll go and have hot wings. And play some arcade games. So, I always associated that as a fun Saturday. (Participant 13)

One participant captured the emotional complexity of growing up around firearms in their community, where exposure to firearm violence coexisted with a sense of enjoyment and familiarity:

You mention the word firearm [to people in the community], and it takes them back, you know, to some form of PTSD, back to something that happened to them, or they lost someone close to them, you know? Someone was shot, someone was robbed, someone was murdered, you know, something like that… So, whenever the topic of firearms or a gun comes up, they immediately retreat back to that space. And although I grew up with those kinds of things, we always just enjoyed firearms, how they looked, how they fired, and stuff like that. (Participant 15)

Participants repeatedly talked about being taught how to properly clean, handle, and use their families’ firearms from a young age. Their families’ firearms were typically stored securely in the home, e.g., in gun safes that only adults could access:

Only adults had the pin or the code [to the gun safe]. That was the standard. I didn’t get the code to the guns in the house until I turned 18. And I think my dad would repeatedly change it, like, every two months, every three months. (Participant 1)

However, not all participants recalled safe storage practices. One participant described how firearms were left unsecured, relying instead on verbal warnings, which proved ineffective, fueling their curiosity and leading them to access the firearms anyway:

Personally, like growing up at home, my dad didn’t own a safe. He had his rifles the closet. He just threatened us, “You better not touch it.” But of course, we did. (Participant 7)

#### Key concept 1.2: introduction to firearms in adulthood

Other participants described first becoming familiar with firearms in adulthood. Their firearm experiences were shaped by a range of motivations. For some, professional roles in the military or law enforcement served as their entry point into firearm use. These participants received formal training as part of their job requirements, which in turn shaped their perceptions of firearm safety and responsibility. Several participants became interested in firearms as adults for hunting, recreation, or due to a general interest in the form and function of different firearms (i.e., a fascination with the physics of firearms). For them, shooting was seen as a hobby or activity that was technical, aesthetic, and enjoyable, e.g.:

I’ve always had an inquisitive nature. I’ve had to know how something works, how it functions, why it functions the way it does. Even with, like, trains, planes, you know, I guess just being curious and just wanting to know why things work the way they do. (Participant 13)

Several participants sought out firearms as adults for personal safety and protection, sometimes after experiencing or witnessing violence or discrimination, either firsthand or indirectly, such as through high-profile law enforcement killings of Black individuals like Tamir Rice.

Regardless of their motivation, most of these participants reported a commitment to learning how to store and use firearms safely, even though several participants had to overcome an initial fear of firearms. They sought out training through concealed carry courses, instruction from family members, or organizations such as the National Rifle Association (NRA), U.S. Concealed Carry Association, NAAGA, and local ranges, with several participants noting the comradery with others that developed during those experiences. Multiple participants pursued certification to become firearm safety instructors themselves for recreational or entrepreneurial reasons. One participant emphasized their motivation to challenge what they viewed as negative stereotypes about Black firearm owners and increase the visibility of responsible firearm ownership as a Black individual:

I know it’s looked upon negatively within the Black community because we’re not looked at the same as gun owners. So, I wanted people to realize that we own our firearms legally. We do take the steps. We do take classes. Everyone is not a criminal. And I said, “I just wanted to bring that to light as well.” A lot of us take it seriously. We actually go train, you know? I want to take us out of that negative [narrative]. (Participant 14)

### Topic 2: why firearms are in the home

#### Key concept 2.1: firearms kept in the home for protection

Most of the participants kept firearms in their homes to protect themselves, their families, and their property. The perceived need for protection through firearm ownership was repeatedly a response to fear – a fear of some external threat that might disrupt their lives. Specific fears included the fear of experiencing racial violence, racism, or racial prejudice, even when the participants had not directly experienced such prejudice, e.g.:

During the last presidential election, some of the supporters of one of the candidates thought that it was okay to run members of [a historically African American fraternal organization] off the road, which I am a member of that [organization]. And so…that was the straw that broke the camel back for me. So, at that time, I took the [fraternal organization] license plate off my car, and I asked my brother to take me to buy a gun so I can protect myself. (Participant 9)

Additional fears included a fear of waiting too long for law enforcement to respond in a time of crisis or exposure to crime, a fear of civil unrest during the COVID-19 pandemic, and fears and suspicions related to prior military service experiences.

Several participants were motivated to acquire firearms following personal experiences with violence, including firearm violence; threats of violence, even at places of religious worship; or crime. One participant described how a home break-in prompted an immediate decision to purchase a firearm, not out of preparation, but as a protective instinct and the urgent need to feel safe:

The first reason that I bought a firearm is a common reason. When I moved, someone broke into the house I was renting, so I was like, “I’m gonna go get a gun.” And that’s what I did. I didn’t do any research. I ended up buying a gun that I have not used, probably since I bought it. I was green with that, so I didn’t really have the knowledge. I didn’t do any research, so I just went out, bought a gun so that you feel, quote, unquote, you know, “safe.” (Participant 10).

Several participants stressed that doing so was their right under the Second Amendment to the U.S. Constitution – their right to purchase firearms, defend themselves, and protect their families. For multiple female participants, firearm ownership was not only about personal safety but also about having the privilege to take their family’s protection into their own hands, much like their own mothers did.

#### Key concept 2.2: firearms kept in the home for work, hobby, and education

Participants also reported keeping firearms in their homes for occupational, recreational, and educational reasons. For example, some participants had law enforcement and security personnel jobs requiring them to carry firearms, while other participants kept firearms in their homes for recreation and hobby, including activities like competitive target shooting, skeet-shooting, and hunting. Multiple male participants were motivated to keep firearms in the home and model responsible ownership for their children. They viewed this as an opportunity to teach their children about firearms in more constructive ways than those sometimes portrayed or experienced in their communities., e.g.:

I tell people all the time, I wouldn’t have trusted myself at 16 with a firearm. Because I was just immature and irresponsible versus my daughter. Me going through my life experiences…made it easier for me to prepare her to be in a house with guns. (Participant 5)

These participants sought to teach their children how to safely handle, clean, and secure firearms in their homes, and, in doing so, demystify firearms and decrease their children’s curiosity about accessing firearms. One participant described their desire to instill an understanding about the danger of firearms, given the ubiquity of firearms in their communities:

For me, making sure that my children are very aware and knowledgeable about not only the firearm itself, but how it operates and the destruction that it can cause in the wrong hands was very, very important. (Participant 4)

### Topic 3: challenges to keeping firearms safe in the home

#### Key concept 3.1: access to firearm safety knowledge and training

Despite being asked about challenges to safely storing firearms in the home, participants discussed a broader lack of – and limited access to – reliable firearm safety knowledge in Black communities as a key challenge to safe firearm storage and keeping firearms safe in the home. Multiple participants expressed their worry that too many Black firearm owners do not fully understand how and when to use a firearm properly, e.g., how to properly load, handle, and shoot their firearms, or store their firearms securely. Of concern for these participants were the potential consequences of acquiring firearms for self-defense without understanding the harm that irresponsible firearm handling can inflict on oneself or others, regardless of whether the firearms are securely stored. For one participant, limited firearm knowledge was the primary challenge to ensuring secure firearm storage and safety in the home; the participant also expressed their view that Black communities should not face unnecessary barriers to firearm ownership and knowledge:

The Bible has a verse in Hosea 4:6, that says, “My people are destroyed for the lack of knowledge.” And I believe that that is the biggest barrier that we have. It should be the responsibility of every gun owner to be properly trained on all the aspects of being a responsible firearm owner. But there’s got to be a medium in there. [Not] everybody needs to go through a million hours of training before they can get a gun. We can send you away to war, but we can’t trust you with a gun and all these other kinds of things. It’s our rights, too. It’s our constitutional rights. (Participant 6)

Participants specifically expressed their concern that there may be limited knowledge about firearm storage and safety devices (e.g., safes and locks) within Black communities. Even when locks are distributed for free by firearm dealers or healthcare entities in Black communities, instruction on how to use the locks is not always provided.

For some participants, limited knowledge of firearm storage and safety practices was not perceived as the firearm owners’ fault; rather, limited firearm safety knowledge was the consequence of inaccessible training or instruction in Black communities, which can lead to inadvertent ignorance about firearm basics, storage, safety, and laws:

Carelessness is you knew better. Ignorance is, you know, I don’t speak this in a derogatory way, but ignorance is just not knowing. (Participant 13)

Additionally, several of these participants described how it can be taboo to talk about firearms in Black communities, which may prevent prospective Black firearm owners from seeking or discovering available storage and safety resources, including locks and other devices and instruction on how to use them. Finally, one participant expressed their concern that concealed carry licensure courses were readily available but focused too much on firearm laws, not firearm mechanics, storage, safety, or use, which may lead to injuries or self-harm:

[They] didn’t teach you anything about a firearm. They taught you the law, that if you hurt someone, you know, if you use your gun or something, it’s a crime, but it did not teach you anything about the firearm. … For me, the concealed carry class should be a one-day class on the laws and regulations and four days of firearms training. You know? Where they’re actually out there and, day one, all they’re doing is taking and cleaning and learning about that particular firearm, where the safeties are, if it has a thumb safety on it versus a trigger safety. Those kinds of things. (Participant 4)

#### Key concept 3.2: opportunity costs – time and money

Participants described various costs as barriers to safe firearm storage in the home. These costs included the price of specific types of firearm storage devices, notably gun safes and biometric safety devices. Reflecting on their interactions with other adults in their community, one participant asserted the following advice:

If you can afford to purchase that gun, you should be able to afford to find a way to store it and lock it. I tell people gun ownership is not cheap. If this is something that you want, you know, you have to consider the expenses that come along with it. (Participant 14)

Additionally, participants discussed the financial burden of maintaining firearm safety practices beyond storage alone, including the cost of firearm safety instruction, insurance, and ammunition for practicing and becoming comfortable with operating one’s firearm, as well as the potential consequences of not being able to afford ongoing training or practice. They worried that, even with initial training, being unable to afford additional firearm safety instruction – or becoming too complacent over time – would negatively affect their attitudes and behaviors on keeping firearms safe in their homes.

Participants also identified the opportunity cost of time. In addition to having the necessary financial resources, having ample time to commit to staying well-informed of best storage and safety practices (e.g., training, mindfulness, and preparation) can be challenging due to competing life priorities. Similarly, several participants who kept firearms in the home for safety described a perceived trade-off between secure storage and rapid access in an emergency. As Black adults, given the fear of an external threat disrupting their lives (e.g., community or racial violence), the ability to quickly access a loaded firearm for self-defense was seen as more important for protecting themselves and their families than adhering to ideal storage practices. For one participant, the trade-off between safe storage and quick access was further complicated by financial constraints, particularly in Black communities facing economic hardship. The participant reflected on the difficult choices families must make when resources are limited and safety is a concern:

There’s not a lot of high earners in this area. And with blue collar towns, you have blue collar wages. And with blue collar wages, and the cost of living going up, you know, you have to make those decisions on how to best secure your family and best take care of your family. (Participant 11)

#### Key concept 3.3: concerns about children gaining access to firearms and the importance of communicating with children about firearms in the home

Although the interview questions did not explicitly ask about children, the participants repeatedly and spontaneously discussed their worry about their children’s curiosity, perceptiveness, and capability of gaining access to their firearms intentionally or unintentionally. As expressed by one parent when reflecting on their household gun safe:

A child’s going to figure it out. That password you think they don’t know. They’re going to figure it out. (Participant 5)

Participants who perceived no other challenges to safely storing their firearms in the home expressed these concerns, as did participants who reported safely storing their firearms, with one participant noting the evolving challenges of preventing children from accessing household firearms as children get older, e.g.:

I don’t think we have any barriers [to safely storing our firearms]. … [But] as long as [they’re] probably like age five or six, the safe is not your problem. By the time they get older, young children are curious. (Participant 1)

Another participant stressed these concerns by referencing conversations with other people in their community, as well as online videos they had encountered, underscoring their worry about underestimating children’s curiosity and ability to access firearms in the home:

I’ve been talking to parents in particular and how they just don’t realize that their child can access their gun. In their mind, “Oh, I put it up high enough. They can’t reach it.” [On YouTube] there is a video of a baby. The parent told the baby that there was something in the cabinet above the refrigerator. And the child scaled that refrigerator like they were Spider-Man. Yeah. And he was still in Pampers. It is like, OK, if this child can get to the top of that refrigerator, what makes you think that your child can’t find your gun? (Participant 9)

Participants also discussed their concern about how to effectively communicate with children in the home about the purpose of firearms and firearm safety devices in ways that demystify firearms and emphasize the danger and consequences of trying to access household firearms. One participant framed this challenge within a broader context, specifically addressing the need to combat cultural messages that, in their view, normalize firearms and potential storage practices in detrimental ways among youth in Black communities, including the portrayal of firearms in video games and music as socially acceptable, fun, and consequence-free. The participant asserted their view that popular media often distorts the Black community’s understanding of firearms, storage, and safety practices at a young age:

It usually comes from what they’ve seen either on TV, heard in some music they were listening to, or a video game. … Unfortunately, you are what you consume, unless you know better. And a lot of people haven’t had that knowledge drilled into them to know that, hey, Call of Duty might not be a good source of firearms knowledge. … I’m not necessarily saying you got to show them, like, a dead body or anything but communicate and show them the harm that could come from accessing a firearm…or not using it safely and things like that. (Participant 13)

Collectively, participants emphasized the importance of proactive communication with children and nurturing a culture of safety in their communities at a young age.

## Discussion

This study addressed an important knowledge gap regarding firearm ownership, storage, and safety among Black adults in the U.S., a population purportedly experiencing increases in firearm ownership yet remaining understudied in firearm safety research. Participants in this study revealed that Black adults’ relationships with firearms appear to be shaped by family traditions, vulnerability, and safety concerns that sometimes extend across generations. Participants described various pathways to firearm access, from childhood introductions through parents who viewed firearms as necessary protection against racial threats, to adult acquisition motivated by experiences of violence, discrimination, or a hobby. While protection emerged as a dominant reason for keeping firearms in homes – especially protection from the fear of racial violence, racism, or racial prejudice – firearms were also maintained for occupational reasons, recreational activities, and educational purposes, particularly to model responsible ownership for their children. Finally, we asked about challenges to safely storing firearms in the home, and three key challenges to household firearm storage safety emerged from the interviews: limited access to firearm safety knowledge and training in Black communities, the financial and time costs associated with safe firearm storage, and concerns about children gaining access to firearms and the importance of communicating with children about firearms in the home. These findings may underscore the need for interventions and initiatives that acknowledge the experiences shaping firearm ownership decisions and household storage safety within Black communities.

Findings from this study appear to be consistent with the broader literature on the motivations for firearm ownership. While the needs and motivations for acquiring firearms documented in our study – including self-protection and recreation – span racial and ethnic identities [7, 8], what is considered “self-protection” for one community may represent something completely different for another. Larger studies at the state and national level have demonstrated that Black individuals may own firearms for self and family protection [8], as well as that exposure to discrimination may be associated with firearm ownership among Black adults [9]. Our qualitative findings suggest that protection from the fear of racial violence, racism, or racial prejudice was particularly motivating among some participants in our study, thus providing additional depth to the literature’s understanding of what “self-protection” means within the context of individuals sharing a Black racial identity. To that end, our findings appear to represent contemporary evidence consistent with the historical Black tradition of arms as chronicled by Johnson (2014) [17]. For example, for generations, many Black Americans have deeply valued their choice to own firearms as a means of not only self-protection but also *self-preservation*, stemming from generations of facing racism, racial prejudice, and exploitation [17]. Lastly, our findings emerged from a sample of participants not primarily characterized by prior experiences with firearm violence (e.g., [10]), or crime, and is thus differentiated from studies specifically focusing on Black individuals, firearms, and issues related to interpersonal violence or crime (e.g., [34–36]). Although important for public health, focusing primarily on the nexus of firearms and criminality or interpersonal violence may fail to capture the full spectrum of firearm-related experiences in Black communities.

Our findings also complement the literature on barriers to safe storage in the home, providing nuance about the experiences of Black adults. Similar to our finding on financial barriers to safe firearm storage (Key Concept 3.2), Khazanov et al.’s (2025) study of primarily white adult military veterans found that the high cost of preferred locking devices was a key barrier to secure storage [37], while other researchers have documented a link between material hardship and storing firearms loaded and unlocked in a large and diverse sample of adults living in households with firearms [38]. Additionally, other studies have reported that the desire for quick firearm access may compromise secure firearm storage in the home; however, the motivations for quick firearm access may differ between demographic groups and/or communities. For instance, in their study of mostly white adults living in rural settings, Ewell Foster et al. (2024) reported that the need for quick access for hunting emerged as a key barrier to secure firearm storage in the home [39], whereas the ability to quickly access a loaded firearm for protection from external threats – again, including the perceived threats of community and racial violence – was seen as more important than adhering to ideal storage practices among participants in our study. Finally, at least one other study has demonstrated participants’ fear of children in the home breaching physical storage devices, thus complicating firearm injury prevention efforts [40]. Though broadly similar to our finding (Key Concept 3.3), participants in our study distinctly elucidated the perceived need to address cultural messages that may, from their perspective, normalize firearms among youth in Black communities in detrimental ways, including the concern that popular media may negatively distort the Black community’s understanding of firearms and firearm safety practices at a young age.

Community-based initiatives can help address the household firearm storage and safety challenges identified by participants in this study. Ideas for such initiatives include partnering with NAAGA or other trusted organizations in the Black firearm community to develop and disseminate educational materials (e.g., posters and social media posts) to provide contact information for firearm instructors, gun ranges, and safety device retailers in each state. Additionally, age-appropriate firearm storage and safety messaging, or a population-centered program to teach parents about safe firearm storage and how to effectively relay firearm safety information to their children, may help address participants’ concerns about how to discuss firearms with children while managing their curiosity and preventing unauthorized access. Such resources could be distributed through partnerships with community leaders and shared in spaces where Black adults feel represented (e.g., faith-based organizations, barbershops, community centers, parent-teacher associations, and Black-owned businesses) to ensure the information reaches those who need it through messengers they trust. New educational materials may also include information on secure firearm storage, loading, and handling techniques and messaging that acknowledges and validates the legitimate fears of racial violence and external threats that motivate firearm ownership among many Black adults. Moreover, these materials should aim to address the tension between needing rapid access to firearms for self-defense and secure storage, offering practical solutions such as affordable storage solutions that maintain security while enabling emergency use (e.g., specific biometric device options), potentially preempting the concern that safe storage practices compromise personal protection.

Although previous studies have suggested that Black adults may be willing to talk to physicians and medical professionals about firearm safety [41, 42], Black adults report being screened for firearm access infrequently in healthcare settings [43]. As such, improving the regularity and quality of firearm safety discussions [44] – and the availability of free safe storage resources [45] – for Black adults in healthcare settings may make the difference between life and death for those experiencing suicide risks. Specifically, findings from this study can be leveraged by healthcare professionals to improve the cultural sensitivity of firearm storage and safety information offered to Black adult patients. This includes incorporating language on family traditions, specific protection motivations, and firearm storage safety challenges experienced by Black firearm owners. Additionally, clinician-focused initiatives could involve working with organizations like NAAGA to train healthcare professionals on population-centered strategies for discussing firearm storage safety with racial/ethnic minority patients, including how to navigate conversations about household firearm storage without triggering defensiveness or reinforcing patients’ fears of adverse consequences for divulging their firearm ownership. For healthcare organizations that provide free locking devices, such partnerships should ensure clinical staff receive training on how to properly demonstrate device usage and discuss locking devices with patients, thereby addressing the participants’ concern that healthcare organizations provide devices without adequate instruction on proper use (Key Concept 3.1).

### Limitations

First, given the use of a rapid qualitative analysis approach, key concepts in this study were identified as *preliminary* or *emerging* through structured summaries in matrix format rather than comprehensive line-by-line coding of full transcripts and thematic analysis. These preliminary key concepts reflect early patterns observed across participants and served as a useful foundation for collaborative team interpretation and dissemination, including timely insights that may help inform firearm safety material development and future research. Nevertheless, the key concepts presented in this paper may not capture the complete nuance, diversity, or range of perspectives present in the data. As such, they should be interpreted with caution, particularly regarding thematic depth and representativeness.

Second, as it is not just the firearm owner who may be at risk if a firearm is stored unsafely, but anyone who may have potential access to it in the household, we intentionally sought to encourage input broadly from Black adults who have access to firearm in their homes. Two participants did not identify as firearm owners themselves but had direct access to – and knowledge of handling – firearms in their homes. No notable differences emerged from the responses of the two participants who did not identify as firearm owners, compared to those who did identify as firearm owners. Prior research has shown that there may be different perceptions of safe firearm storage practices, personal firearm injury risk, and the acceptability of interventions like firearm safety counseling between owners and non-owners [46–48]. However, our study was not designed to investigate such differences. To build on this work and focus on the safety of all members of households with firearms in Black communities, future studies should investigate differences in the perceptions of firearm safety and risk specifically among Black adults who live in homes with access to firearms but who do not own firearms themselves.

Third, the 5% threshold for new information was a subjective benchmark [26]. There is no guarantee that saturation is reached when meeting such a threshold. Rather, the threshold represents a transparent way to present the potential saturation of information generated through this rapid qualitative analysis to the reader.

Fourth, the interviewer’s (EG) racial identity differed from that of the participants. Notwithstanding his commitment to cultural humility during the interviews, and his knowledge of firearms, EG was aware of his status as an “outsider” [49] to the community he was interviewing, which may have affected the participants’ openness, comfort, and responses to the interview questions. For this reason, EG was highly attentive to the power relations inherent in this work and engaged in the reflexive activities noted earlier to attempt to mitigate bias.

Fifth, although positivist concepts like generalizability were not a goal of this work, readers should be attentive when considering the transferability of the insights generated through this study to the experiences of other Black adults. This may be due to our small sample size or sampling strategy (e.g., word-of-mouth referrals from likeminded participants), the limited information collected from the participants by which to compare to other individuals, or the fact that the insights the participants shared reflected their lived realities and no one else’s. Nevertheless, we attempted to mitigate transferability concerns by providing the reader with a detailed description of the participants’ experiences and perceptions relevant to the study objective [50] (including the use of detailed illustrative quotes) and by incorporating relatively wide geographic variation in the sample [20]. As is expected in qualitative inquiry, other studies, contexts, or individuals may yield different insights; nevertheless, the information shared by participants in this study complement the existing literature by offering depth and nuance regarding the experiences of Black adults who have access to firearms in their homes.

Finally, depending on the state of residence, specific public policies such as secure storage laws or child-access prevention laws may have affected the participants’ firearm storage behaviors and subsequent challenges to safely storing firearms in their homes. Of note, public policies were not a topic of the interviews, and specific policies were rarely mentioned by the interviewees, which may in part be related to previous research suggesting many firearm owners are not aware of whether they lived in states with a specific type of child-access prevention law [51]. 

## Conclusions

This study provides new insights into the factors shaping firearm ownership and perceived challenges to keeping firearms safe in the home among a sample of Black adults in the U.S. Participants in this study revealed that firearm ownership may be rooted in historical and contemporary experiences, family traditions, and safety concerns, while household firearm storage safety is challenged by limited access to firearm safety knowledge and training, financial barriers, and concerns about children gaining access to firearms and the importance of communicating with children about firearms in the home. Although not necessarily generalizable, our findings may help inform community-driven and clinician-focused initiatives that acknowledge the context of Black firearm ownership while promoting evidence-based safety practices. As firearm ownership has purportedly increased among Black adults in recent years, public health efforts should move beyond one-size-fits-all approaches to create population-centered programs that respect the rights, values, and lived experiences of Black firearm owners while working to prevent firearm injuries and deaths in Black communities.

## Data Availability

The summarized data generated and/or analysed and presented in this paper (e.g., key concepts) will also be available in the Harvard Dataverse repository: https://dataverse.harvard.edu/dataverse/goldsteinK18.

## Abbreviations

CALM: Counseling on access to lethal means
IRB: Institutional review board
NAAGA: National african american gun association
NRA: National rifle association
